# Antibiotic resistance in uropathogens across northern Australia 2007–20 and impact on treatment guidelines

**DOI:** 10.1093/jacamr/dlab127

**Published:** 2021-08-14

**Authors:** Will Cuningham, Shalinie Perera, Sonali Coulter, Graeme R Nimmo, Trent Yarwood, Steven Y C Tong, Teresa M Wozniak

**Affiliations:** 1 Menzies School of Health Research, Charles Darwin University, Darwin, Northern Territory, Australia; 2 Western Diagnostic Pathology, Western Australia, Australia; 3 Prevention Division, Department of Health, Medication Services Queensland, Queensland, Australia; 4 Central Laboratory, Pathology Queensland, Queensland, Australia; 5 Griffith University School of Medicine, Queensland, Australia; 6 Antimicrobial Use and Resistance in Australia Project, Australian Commission for Safety and Quality in Healthcare, Canberra, Australian Capital Territory, Australia; 7 Cairns Hospital, Cairns, Queensland, Australia; 8 Rural Clinical School, University of Queensland, Brisbane, Queensland, Australia; 9 College of Medicine and Dentistry, James Cook University, Townsville, Queensland, Australia; 10 Victorian Infectious Diseases Service, The Royal Melbourne Hospital at the Peter Doherty Institute for Infection and Immunity, Melbourne, Australia; 11 Department of Infectious Diseases, The University of Melbourne at the Peter Doherty Institute for Infection and Immunity, Melbourne, Victoria, Australia

## Abstract

**Background:**

Urinary tract infections are common and are increasingly resistant to antibiotic therapy. Northern Australia is a sparsely populated region with limited access to healthcare, a relatively high burden of disease, a substantial regional and remote population, and high rates of antibiotic resistance in skin pathogens.

**Objectives:**

To explore trends in antibiotic resistance for common uropathogens *Escherichia coli* and *Klebsiella pneumoniae* in northern Australia, and how these relate to current treatment guidelines in the community and hospital settings.

**Methods:**

We used data from an antibiotic resistance surveillance system. We calculated the monthly and yearly percentage of isolates that were resistant in each antibiotic class, by bacterium. We analysed resistance proportions geographically and temporally, stratifying by healthcare setting. Using simple linear regression, we investigated longitudinal trends in monthly resistance proportions and correlation between community and hospital isolates.

**Results:**

Our analysis included 177 223 urinary isolates from four pathology providers between 2007 and 2020. Resistance to most studied antibiotics remained <20% (for *E. coli* and *K. pneumoniae*, respectively, in 2019: amoxicillin/clavulanate 16%, 5%; cefazolin 17%, 8%; nitrofurantoin 1%, 31%; trimethoprim 36%, 17%; gentamicin 7%, 2%; extended-spectrum cephalosporins 8%, 5%), but many are increasing by 1%–3% (absolute) per year. Patterns of resistance were similar between isolates from community and hospital patients.

**Conclusions:**

Antibiotic resistance in uropathogens is increasing in northern Australia, but treatment guidelines generally remain appropriate for empirical therapy of patients with suspected infection (except trimethoprim in some settings). Our findings demonstrate the importance of local surveillance data (HOTspots) to inform clinical decision making and guidelines.

## Introduction

Urinary tract infections (UTIs) are common infections predominantly caused by Gram-negative Enterobacterales and have a substantial health and economic impact in both the community and hospital setting.^1–5^ The prevalence of healthcare-associated UTIs treated in Australian hospitals has been estimated at between 1%–2%, increasing the patient length of stay by 3–5 days.^6^^,^^7^ UTIs caused by *Escherichia coli* are consistently the most frequently occurring infections in Australian hospitals (7.85 episodes per 1000 patient days) and account for approximately 7% of antibiotic prescriptions (fourth most common indication).^8^^,^^9^

Antibiotic resistance in UTI-causing organisms is common and of most concern in Australia are extended-spectrum β-lactamases (ESBLs), carbapenemases, aminoglycoside-modifying enzymes and ribosomal methylases.^4^^,^^8^ Resistant infections can further increase inpatient length of stay and associated costs.^9^ There is evidence that resistance is more common in the hospital setting compared with the community, is geographically variable and is increasing over time.^10^^,^^11^ Australian data shows that by global standards the prevalence of resistance in general is relatively low, but is considerably higher in the surrounding Asia-Pacific region.^8^^,^^12–14^ Even so, resistance in uropathogens in Australia is increasing, commonly to β-lactams, fluoroquinolones and trimethoprim/sulfamethoxazole.^8^

Given that recommended treatments are often empirical,^15–18^ better knowledge of the common causative pathogens of UTIs and local resistance patterns is essential in determining appropriate therapy, thereby minimizing the risk of increasing resistance.^19^ This is especially relevant in northern Australia since it is a sparsely populated region with a substantial regional and remote population whose access to healthcare (particularly hospitals) is limited. For example, in the Northern Territory (NT) 40% of the population live remotely, of whom 58% are Aboriginal and/or Torres Strait Islander.^20^ Furthermore, the infectious disease burden in northern Australia is high relative to other parts of Australia and compounded by increases in antibiotic resistance.^21–25^*Staphylococcus aureus* demonstrates an increasing prevalence of resistance to common β-lactam antibiotics in northern Australia over time (1993: 7%, 2012: 24%) and at levels much higher than elsewhere in Australia.^8^^,^^26^

There are limited published data on UTI antibiotic resistance epidemiology in northern Australia. We therefore aimed to explore trends in antibiotic resistance for common uropathogenic organisms *E. coli* and *Klebsiella pneumoniae*, and how these relate to current treatment guidelines in the community and hospital settings (Table 1). We hypothesized an increase in resistance rates over time and variation by region.

**Table 1.** Common syndromes associated with UTIs and recommended antibiotics

Syndrome		Route	Antibiotic		
			Therapeutic Guidelines^a^	CARPA^b^	PCCM^c^
Cystitis		Oral	amoxicillin or ampicillin	amoxicillin/ampicillin (pregnant women only^d^)	
			amoxicillin/clavulanate	**amoxicillin/clavulanate** (children only)	**amoxicillin/clavulanate** (pregnant women only)
			cefalexin^e^	**cefalexin** (excl. children)	cefalexin^e^
			ciprofloxacin (excl. pregnant women)		
			fosfomycin (excl. pregnant women and children)		
			**nitrofurantoin** (excl. children)	**nitrofurantoin** (pregnant women only)	nitrofurantoin^e^ (excl. women near delivery)
			norfloxacin (excl. pregnant women)		
			**trimethoprim** (incl. women in 2nd/3rd trimester)	**trimethoprim** (excl. pregnant women and children)	**trimethoprim** (excl. pregnant women)
			trimethoprim/sulfamethoxazole (excl. pregnant women, empirical for children)	trimethoprim/sulfamethoxazole^e^ (children only)	
Pyelonephritis	non-severe	Oral	amoxicillin/ampicillin		
			**amoxicillin/clavulanate** (not empirical for pregnant women)	**amoxicillin/clavulanate** (children only)	
			cefalexin (empirical for children)	**cefalexin** (adults only)	cefalexin^e^
			ciprofloxacin^e^ (excl. pregnant women)		
			trimethoprim (incl. women in 2^nd^/3^rd^ trimester, empirical for children)	**trimethoprim** (adults only)	**trimethoprim** (excl. pregnant women)
			trimethoprim/sulfamethoxazole^e^ (excl. pregnant women, empirical for children)	trimethoprim/sulfamethoxazole^e^ (children only)	
	severe	IV	cefotaxime^e^		
			ceftriaxone^e^	**ceftriaxone** (excl. children)	**ceftriaxone** (pregnant women only)
			**gentamicin (+amoxicillin or ampicillin)**	**gentamicin (+amoxicillin or ampicillin)** (children only)	**gentamicin (+amoxicillin or ampicillin)** (excl. pregnant women)
Prophylaxis		Oral	cefalexin	cefalexin (pregnant women only)	
			nitrofurantoin (excl. women near delivery)		
			trimethoprim (excl. pregnant women)		
			trimethoprim/sulfamethoxazole (children only)		
Sepsis		IV	cefotaxime^e^		
			ceftriaxone^e^		**ceftriaxone** (pregnant women only)
			**gentamicin (+amoxicillin or ampicillin)**		
			meropenem		

Antibiotics recommended as empirical therapy are shown in bold font.

a Electronic Therapeutic Guidelines—Antibiotics (eTG).^16^ Recommended against ESBLs: amoxicillin/clavulanate, fosfomycin, meropenem, nitrofurantoin.

b Remote Primary Health Care Manuals, Standard Treatment Manual (7th edition).^15^

c Primary Clinical Care Manual 10th edition 2019.^17^ Note: specific antibiotics not included for children.

d Group B *Streptococcus* positive.

e Alternative if first-line treatment cannot be used (e.g. penicillin allergy or contraindiction).

## Methods

### Study setting

**Figure 1.**
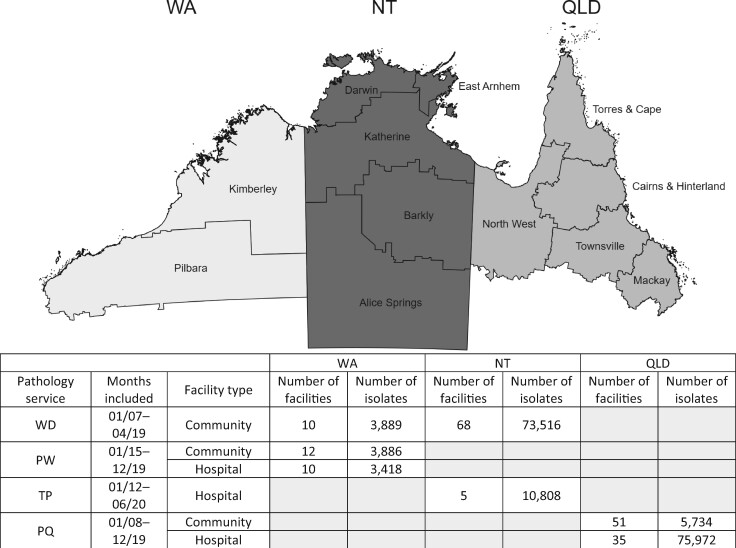
Map of northern Australia and regions represented in dataset, and summary of data sources. WA, Western Australia (Kimberley and Pilbara); NT, Northern Territory; QLD, far north Queensland; WD, Western Diagnostic Pathology; PW, PathWest; TP, Territory Pathology; PQ, Pathology Queensland. The following antibiotics were not included in pathology datasets: (WA, community: gentamicin, trimethoprim/sulfamethoxazole; hospital: cefazolin, ceftazidime, ceftriaxone, ciprofloxacin, gentamicin, trimethoprim/sulfamethoxazole); (NT, community: nitrofurantoin, trimethoprim, trimethoprim/sulfamethoxazole); (QLD, community: nitrofurantoin, trimethoprim, trimethoprim/sulfamethoxazole; hospital: nitrofurantoin, trimethoprim, trimethoprim/sulfamethoxazole).

The data used in this analysis were collected as part of antibiotic resistance surveillance called HOTspots.^27^ HOTspots sources antibiotic susceptibility data from clinical isolates tested by the major pathology providers in northern Australia (Figure 1). In Western Australia (WA), participating pathology providers (Western Diagnostic Pathology and PathWest) include non-hospital healthcare facilities (hereafter referred to as community healthcare facilities), and public hospitals (PathWest). In the NT, participating pathology providers include all public hospitals (Territory Pathology) and all community healthcare facilities (Western Diagnostic Pathology). In Queensland (QLD), participating pathology providers include all public hospitals and a proportion of community healthcare facilities (Pathology Queensland). In this study we define northern Australia as the entire NT and the area above the Tropic of Capricorn in WA and QLD (Figure 1). We divided each jurisdiction into regions based on classification by the Australian Bureau of Statistics (Statistical Area Level 3), with populations ranging from 30 000 to 130 000 people.^20^

### Microbiological data

We used all *E. coli* and *K. pneumoniae* isolates from urinary specimens and their corresponding antibiotic susceptibilities, covering the period from January 2007 to June 2020. Almost all specimens were urine (95%) but also included specimens from the urinary tract (including kidney aspirates, fluid discharge and urethral swabs). No clinical data were available.

Participating pathology providers are accredited under regularly audited national testing guidelines (National Association of Testing Authorities) and are members of the National Quality Assurance and Quality Control programme run by the Royal College of Pathologists of Australasia External Quality Control Assurance programme. Susceptibility testing in the included laboratories was done using a combination of VITEK 2 (bioMérieux) and disc-diffusion techniques. Western Diagnostics and PathWest provided CLSI-interpreted values (Susceptible, Intermediate and Resistant). MICs were provided by Territory Pathology, to which we applied the 2017 CLSI M100-S27 Performance Standards for Antimicrobial Susceptibility Testing. Pathology Queensland provided CLSI-interpreted values from 2008 to June 2012, and EUCAST-interpreted values from July 2012 onwards. All MICs were interpreted using the current breakpoints at the time of data delivery. We defined resistance as a ‘Resistant’ susceptibility result (intermediate was considered to be susceptible).

Based on clinical importance, treatment guideline recommendations (Table 1) and available data,^15–17^ our analysis focused on resistance in five antibiotic classes: β-lactamase inhibitor plus penicillin combinations (amoxicillin/clavulanate), first-generation cephalosporins [cefazolin (used to infer cefalexin resistance)], fluoroquinolones (resistance to ciprofloxacin and/or norfloxacin), aminoglycosides (gentamicin) and extended-spectrum cephalosporins (ESCs) (resistance to ceftriaxone and/or ceftazidime). Supplementary analyses also included ampicillin, nitrofurantoin, trimethoprim and trimethoprim/sulfamethoxazole.

### Statistical analyses

We used Stata 16.1 and R (via RStudio 1.3) to clean and analyse the data.^28^^,^^29^ We calculated the monthly and yearly percentage of isolates that were resistant in each antibiotic class, by bacterium. We mapped resistance proportions by region (for 2019, the most recent year with data from all pathology providers) and plotted them over time using locally weighted regression, stratifying by jurisdiction (WA versus NT versus QLD) and healthcare setting (community versus hospital). Using simple linear regression, we investigated longitudinal trends in monthly resistance proportions and correlation between community and hospital isolates. Months with fewer than ten isolates were excluded and only the first isolate per individual per year was included. We excluded antibiotics that were tested in <75% of isolates.

### Ethics approval

This project was granted ethics approval from the Human Research Ethics Committee of the Northern Territory Department of Health and Menzies School of Health Research (HREC-2018-3084) as well as the Queensland Health Public Health Act 2005 (Section 280). All data were analysed in compliance with the requirements of the National Statement on Ethical Conduct in Human Research (2007).

## Results

### Descriptive characteristics

Our analysis included 177 223 urinary isolates [Western Diagnostics (WA/NT): 77 405; PathWest (WA): 7304; Territory Pathology (NT): 10 808; Pathology Queensland (QLD): 81 706] (Figure 1). Most of these were *E. coli* isolates (86%, *n = *154 387; *K. pneumoniae*: 14%, *n = *24 442). The median age at first isolate collected was 41 years (IQR: 24–64) and 86% (*n = *92 240) were female (age/sex not available from all pathology providers).

### Geographical variation of antibiotic resistance in uropathogens, 2019

**Figure 2.**
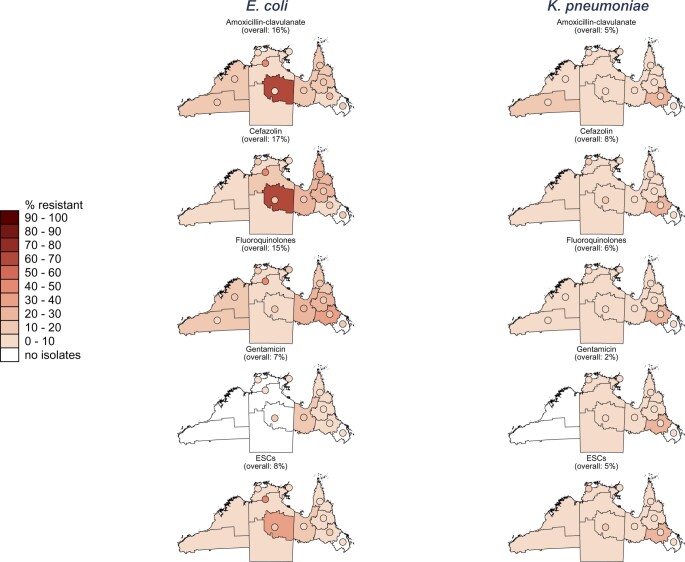
Proportion of isolates resistant to five antibiotics/antibiotic groups in 2019, by region and healthcare setting [community or hospital (displayed as circles)]. ESCs, extended-spectrum cephalosporins (resistance to ceftriaxone or ceftazidime); fluoroquinolones, resistance to ciprofloxacin or norfloxacin (only norfloxacin in WA hospitals). Regions (i.e. community healthcare facilities) with <30 isolates: [WA, all regions (*E. coli* and *K. pneumoniae*, cefazolin and ESCs)]; [NT, all regions (*E. coli* and *K. pneumoniae*, all antibiotics)]; [QLD, Cairns & Hinterland (*E. coli* and *K. pneumoniae*, all antibiotics); North West (*K. pneumoniae*, all antibiotics); Townsville (*E. coli* and *K. pneumoniae*, all antibiotics)]. Hospitals with <30 isolates: [WA, Pilbara (*K. pneumoniae*, amoxicillin/clavulanate and fluoroquinolones)]; [NT: East Arnhem (*K. pneumoniae*, all antibiotics); Katherine (*E. coli*, all antibiotics); Barkly (*K. pneumoniae*, all antibiotics)].

Overall, resistance to all the studied antibiotic classes was higher in *E. coli* isolates compared with *K. pneumoniae* in 2019 (Figure 2). Highest overall (community and hospital combined) resistance in *E. coli* isolates was to ampicillin [Figure S1 is available as Supplementary data at *JAC-AMR* Online; WA: 63%, NT: 66%, QLD: 47% (*P < *0.05 for each pairwise combination)], followed by trimethoprim [Figure S1; WA: 33%, NT: 38% (*P < *0.01)], cefazolin [Figure 2; NT: 12%, QLD: 19% (*P* < 0.001)], amoxicillin/clavulanate [Figure 2; WA: 17% (*P < *0.001 compared with the NT), NT: 5%, QLD: 18% (*P < *0.001 compared with the NT)] and fluoroquinolones [Figure 2; WA: 12%, NT: 11%, QLD: 17% (*P < *0.001 compared with WA and the NT)]. Overall resistance in *K. pneumoniae* isolates was less than 10% for all studied antibiotic classes, except for trimethoprim/sulfamethoxazole in NT hospitals which was 16% (nitrofurantoin and trimethoprim both also >10%, however <30 isolates were tested for each). *E. coli* resistance to trimethoprim/sulfamethoxazole was high in NT hospital isolates (36%) but we could not compare this with other jurisdictions since trimethoprim/sulfamethoxazole susceptibility data was not available.

Resistance differed within jurisdictions as well as between jurisdictions, but the low numbers of isolates tested limited statistical comparisons (especially in WA and the NT).

### Temporal trends of antibiotic resistance in uropathogens

**Figure 3.**
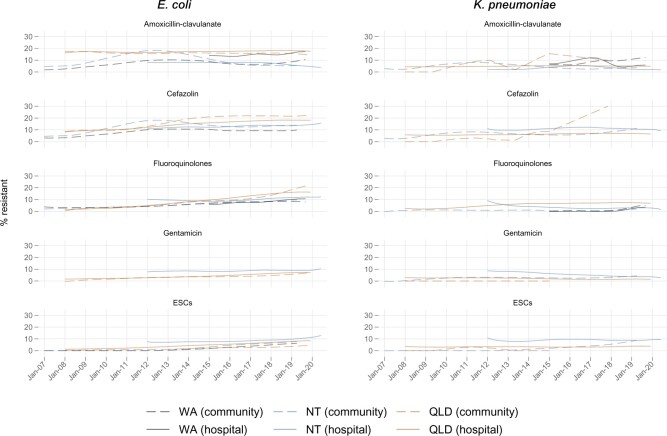
Proportion of isolates resistant to five antibiotics/antibiotic groups over time (smoothed using locally weighted regression), by jurisdiction and healthcare setting. ESCs, extended-spectrum cephalosporins (resistance to ceftriaxone or ceftazidime); fluoroquinolones, resistance to ciprofloxacin or norfloxacin (only norfloxacin in WA hospitals). **Non-significant changes:** Community [(WA: *E. coli*, amoxicillin/clavulanate and cefazolin and fluoroquinolones and ESCs, *K. pneumoniae*, fluoroquinolones); (NT: *E. coli*, amoxicillin/clavulanate; *K. pneumoniae*, fluoroquinolones); (QLD: *E. coli*, amoxicillin/clavulanate; *K. pneumoniae*, amoxicillin/clavulanate and fluoroquinolones)]; Hospital [(WA: *E. coli*, amoxicillin/clavulanate; *K. pneumoniae*, amoxicillin/clavulanate and fluoroquinolones); (NT: *E. coli*, gentamicin; *K. pneumoniae*, amoxicillin/clavulanate and cefazolin and ESCs); (QLD: *K. pneumoniae*, amoxicillin/clavulanate and ESCs)]. **Significant decreases:** Hospital [(NT: *E. coli*, amoxicillin/clavulanate; *K. pneumoniae*, fluoroquinolones and gentamicin); (QLD: *K. pneumoniae*, gentamicin)].

Significant increases in resistance over time were common for *E. coli* and *K. pneumoniae* in both the community and hospital settings (Tables S1 and S2, Figure 3, Figures S2 and S3). Most of these increases ranged from 0.1% to 2.6% (absolute) per year (Table S2). Combining hospital and community isolates, *E. coli* resistance to fluoroquinolones in QLD had the largest increase [1.5% per year (2008: 2%, 2019: 17%); *P < *0.001]. There was also a large increase in *E. coli* resistance to trimethoprim [1.5% per year (2012: 31%, 2020: 42%); *P < *0.001] and trimethoprim/sulfamethoxazole [1.2% per year (2012: 30%, 2020: 38%); *P < *0.001] in NT hospital isolates (Figure S3). There was an increase in *K. pneumoniae* resistance to cefazolin in QLD community isolates [2.6% per year (2008: 0%, 2017: 30%); *P < *0.01], but there was a low number of isolates tested and a low number of available data points (11 months across 10 years).

There were some differences between trends in community and hospital isolates. In WA, *E. coli* resistance to fluoroquinolones increased significantly in hospital isolates but not in community isolates. Conversely, *K. pneumoniae* resistance to amoxicillin/clavulanate increased significantly in WA community isolates but not in hospital isolates; this was also seen for cefazolin and ESCs in the NT. The only other increase in resistance to amoxicillin/clavulanate was for *E. coli* in QLD hospital isolates, and this was only an absolute increase of <2% over 12 years. Furthermore, *K. pneumoniae* resistance to ESCs did not change over time in NT and QLD hospital isolates.

There were some significant decreases in resistance. *K. pneumoniae* resistance to gentamicin in NT and QLD hospital isolates decreased, however resistance in NT community isolates increased. *E. coli* resistance to amoxicillin/clavulanate and *K. pneumoniae* resistance to fluoroquinolones and nitrofurantoin decreased in NT hospital isolates.

Generally, the differences between resistance in community isolates and hospital isolates were minimal (<10%). Positive correlation between resistance in community and hospital isolates was evident (Tables S3 and S4, Figures 2 and 3, Figures S1 and S3), especially for *E. coli* isolates in QLD [coefficients from linear regressions—cefazolin: 1.17 (95% CI: 0.91–1.44); fluoroquinolones: 0.87 (0.71–1.03); gentamicin: 0.68 (0.43–0.94); ESCs: 0.44 (0.29–0.59)]. There was a strong correlation for fluoroquinolone resistance in *E. coli* and *K. pneumoniae* isolates in all three jurisdictions. None of the negative correlations were significant, except for *E. coli* ampicillin resistance in QLD (Figure S3).

## Discussion

### National and international context

Northern Australia is a geographically vast area with demographic, climatic and healthcare differences both within the region and compared with the rest of Australia (i.e. below the Tropic of Capricorn). Despite these differences, in general, variation in uropathogen antibiotic resistance between northern and southern Australia is minimal.^8^ Some notable exceptions are resistance to trimethoprim (37% versus 25%) and trimethoprim/sulfamethoxazole (38% versus 20%) in *E. coli* isolates, which are both higher in northern Australia.

The differences between northern and southern Australia show that it is important to ensure local antibiotic treatment guidelines are up to date and informed by local antibiotic susceptibility data. Furthermore, it is more informative to interpret temporal trends rather than point prevalence (e.g. a yearly cross-sectional estimate) due to fluctuations in resistance (e.g. due to seasonal variation).^11^^,^^30–34^

Compared with global rates of resistance for *E. coli*/*K. pneumoniae* and in the context of the Asia-Pacific region where ESC and fluoroquinolone resistance is often in excess of 30% and 50% respectively, northern Australia is in an enviable position.^10^^,^^12–14^^,^^35^ However, resistance is increasing steadily. Between 2015 and 2017, *E. coli* fluoroquinolone resistance has risen in Australia from 3rd lowest (10.5%) to 6th lowest (14.4%) compared with European Union countries.^8^ More narrow-spectrum agents such as cefalexin remain a better option than fluoroquinolones, which continue to be restricted for use in Australia.^36–38^

### Implications for guidelines

#### Community setting

In general, *E. coli* and *K. pneumoniae* resistance proportions remain at a clinically manageable level. Overall, only 10% of *E. coli* isolates were resistant to all first-line oral agents [WA (Western Diagnostics data only): 5%; NT: 8%; QLD: 11%]. First-line agents cefalexin and amoxicillin/clavulanate (resistance ≤20%) are still reasonable treatment options for conditions such as cystitis and non-severe pyelonephritis. However, the increase in *E. coli* cefalexin resistance, especially in QLD from 9% to 19% between 2008 and 2019, should be monitored closely. Furthermore, cefalexin is one of the most commonly used antibiotics in Australia and use is often non-compliant with guidelines.^8^^,^^39^ Promisingly, a pilot audit of antimicrobial use in remote primary healthcare in northern Australia indicates that the antimicrobial therapy for UTIs is often clinically appropriate and guideline-compliant.^40^

All three treatment guidelines used in northern Australia recommend trimethoprim as a first-line agent,^15–17^ but our data show that *E. coli* resistance to this antibiotic is increasing above 30% in both WA and the NT (QLD data not available). Preliminary data indicate that trimethoprim is the most common antimicrobial used to treat UTIs in remote northern Australian primary healthcare,^40^ however, nationally in 2017 only 45% of female adults with a UTI received trimethoprim, indicating that cefalexin is often used preferentially.^8^ In northern Australia, where there is relatively low cefalexin resistance (e.g. <15%) and relatively high trimethoprim resistance (e.g. >25%), cefalexin may be preferred.

Similarly, *E. coli* resistance to trimethoprim/sulfamethoxazole in the NT has increased from 30% to 40% over the past 5 years. This increase may be associated with recent guidelines recommending trimethoprim/sulfamethoxazole as first-line treatment for skin and soft tissue infections (SSTI), particularly due to methicillin-resistant *S. aureus*.^41^^,^^42^ Regions in northern Australia with a higher prevalence of SSTIs appear to also have higher uropathogen resistance rates to trimethoprim/sulfamethoxazole.^8^^,^^26^^,^^43^ Trimethoprim/sulfamethoxazole is mainly indicated for use in children, and while our data would need to be stratified into age groups for more detailed commentary, this recommendation may need to be revised. Fluoroquinolone resistance is also increasing, especially in *E. coli* isolates in QLD, despite restrictions on use.

#### Hospital setting

In the hospital setting, current recommendations for the use of gentamicin (*E. coli*: 7% resistant; *K. pneumoniae*: 2% resistant in 2019) and ceftriaxone (*E. coli*: 8% resistant; *K. pneumoniae*: 5% resistant in 2019) to treat severe UTIs and complications such as pyelonephritis and sepsis are still appropriate (although there have been increases *E. coli* resistance).

### Limitations

These data are comprised of susceptibility tests from pathology providers who use either CLSI or EUCAST standards. This is an ongoing challenge in ensuring data are comparable, however the differences between these two methodologies for the organisms and antibiotics included in this study are minimal and unlikely to impact the overall conclusions substantively.

We classified isolates into community and hospital based on the facility at which the specimen was collected. In the remote region of northern Australia, some hospitals are more akin to a primary healthcare facility in a metropolitan setting than a tertiary hospital. Furthermore, since we had no clinical data or information on patient history, we could not define community-acquired or healthcare-associated infections (or indeed distinguish between colonization and infection). The literature suggests that approximately 50% of UTIs treated in hospital are community-acquired.^6^^,^^44^^,^^45^

Finally, the Australian Therapeutic Guidelines recommend that urine culture may not be necessary for non-pregnant women presenting with their first UTI.^16^ Other regional guidelines do not make this distinction, however, in a remote setting, sending a sample for microbiological analysis is more difficult and so might be less common for patients at their first presentation.^15^^,^^17^ Since patients with recurrent UTIs are more likely to be infected with an antibiotic-resistant organism, it is possible the resistance proportions we have observed are an overestimation.

### Conclusions

In northern Australia resistance in uropathogens is slowly increasing, but in most cases, guidelines remain appropriate for empirical therapy. Cefalexin, nitrofurantoin or amoxicillin/clavulanate might be better treatment options in settings where trimethoprim resistance is high (e.g. >25%) and resistance to these other first-line agents is relatively low (e.g. <15%). These comparably low resistance rates are in sharp contrast to neighbouring Asia-Pacific countries and the resistance profile of other problematic pathogens in northern Australia such as *S. aureus*.

Our findings demonstrate the importance of this antibiotic resistance surveillance system (HOTspots)^27^ as an adjunct to clinical guidelines for health professionals in northern Australia. Crucially, HOTspots provides detail at a local level, capturing variations in antibiotic resistance between regions and healthcare settings. Planned additions to this dataset such as age and sex will further enrich this resource.

## Supplementary Material

dlab127_Supplementary_Data

## References

[1] Tandogdu Z, Wagenlehner FME. Global epidemiology of urinary tract infections. Curr Opin Infect Dis 2016; 29: 73–9. 26694621 10.1097/QCO.0000000000000228

[2] Foxman B. Epidemiology of urinary tract infections: incidence, morbidity, and economic costs. Dis Mon 2003; 49: 53–70. 12601337 10.1067/mda.2003.7

[3] Laupland K, Ross T, Pitout J et al Community-onset urinary tract infections: a population-based assessment. Infection 2007; 35: 150. 17565455 10.1007/s15010-007-6180-2

[4] Öztürk R, Murt A. Epidemiology of urological infections: a global burden. World J Urol 2020; 38: 2669–79. 31925549 10.1007/s00345-019-03071-4

[5] European Centre for Disease Prevention and Control. Point prevalence survey of healthcare-associated infections and antimicrobial use in European long-term care facilities. April–May 2013. 2014. https://www.ecdc.europa.eu/en/publications-data/point-prevalence-survey-healthcare-associated-infections-and-antimicrobial-use-2.

[6] Gardner A, Mitchell B, Beckingham W et al A point prevalence cross-sectional study of healthcare-associated urinary tract infections in six Australian hospitals. BMJ Open 2014; 4: e005099. 10.1136/bmjopen-2014-005099 PMC4120374 25079929

[7] Mitchell BG, Ferguson JK, Anderson M et al Length of stay and mortality associated with healthcare-associated urinary tract infections: a multi-state model. J Hosp Infect 2016; 93: 92–9. 26944900 10.1016/j.jhin.2016.01.012

[8] Australian Commission on Safety and Quality in Health Care (ACSQHC). AURA 2019: third Australian report on antimicrobial use and resistance in human health. 2019. https://www.safetyandquality.gov.au/sites/default/files/2019-06/AURA-2019-Report.pdf.

[9] Wozniak TM, Bailey EJ, Graves N. Health and economic burden of antimicrobial-resistant infections in Australian hospitals: a population-based model. Infect Control Hosp Epidemiol 2019; 40: 320–7. 30887942 10.1017/ice.2019.2

[10] Fasugba O, Gardner A, Mitchell BG et al Ciprofloxacin resistance in community- and hospital-acquired *Escherichia coli* urinary tract infections: a systematic review and meta-analysis of observational studies. BMC Infect Dis 2015; 15: 545. 26607324 10.1186/s12879-015-1282-4 PMC4660780

[11] Fasugba O, Mitchell BG, Mnatzaganian G et al Five-year antimicrobial resistance patterns of urinary *Escherichia coli* at an Australian Tertiary Hospital: time series analyses of prevalence data. PLoS One 2016; 11: e0164306. 27711250 10.1371/journal.pone.0164306 PMC5053592

[12] Lu PL, Liu YC, Toh HS et al Epidemiology and antimicrobial susceptibility profiles of Gram-negative bacteria causing urinary tract infections in the Asia-Pacific region: 2009–2010 results from the Study for Monitoring Antimicrobial Resistance Trends (SMART). Int J Antimicrob Agents 2012; 40: S37–43. 22749057 10.1016/S0924-8579(12)70008-0

[13] Jean SS, Coombs G, Ling T et al Epidemiology and antimicrobial susceptibility profiles of pathogens causing urinary tract infections in the Asia-Pacific region: results from the Study for Monitoring Antimicrobial Resistance Trends (SMART), 2010-2013. Int J Antimicrob Agents 2016; 47: 328–34. 27005459 10.1016/j.ijantimicag.2016.01.008

[14] Rogers BA, Ingram PR, Runnegar N et al Community-onset *Escherichia coli* infection resistant to expanded-spectrum cephalosporins in low-prevalence countries. Antimicrob Agents Chemother 2014; 58: 2126–34. 24468775 10.1128/AAC.02052-13 PMC4023745

[15] Centre for Remote Health. CARPA Standard Treatment Manual (7th edition). 2017. https://healthinfonet.ecu.edu.au/healthinfonet/getContent.php?linkid=592687&title=CARPA+standard+treatment+manual%3A+a+clinic+manual+for+primary+health+care+practitioners+in+remote+and+Indigenous+health+services+in+central+and+northern+Australia.

[16] Therapeutic Guidelines Limited. eTG complete. 2015. https://tgldcdp.tg.org.au/etgAccess.

[17] Queensland Health. Primary Clinical Care Manual 10th edition. 2019. https://www.health.qld.gov.au/rrcsu/clinical-manuals/primary-clinical-care-manual-pccm.

[18] Gupta K, Hooton TM, Naber KG et al International clinical practice guidelines for the treatment of acute uncomplicated cystitis and pyelonephritis in women: a 2010 update by the Infectious Diseases Society of America and the European Society for Microbiology and Infectious Diseases. Clin Infect Dis 2011; 52: e103–20. 21292654 10.1093/cid/ciq257

[19] Collignon P. MJA practice essentials; 11. Antibiotic resistance. Med J Aust 2002; 177: 325–9. 12225283 10.5694/j.1326-5377.2002.tb04794.x

[20] Australian Bureau of Statistics. 3218.0 - Regional Population Growth, Australia. 2018. https://www.abs.gov.au/ausstats/abs@.nsf/Previousproducts/3218.0Main%20Features12016-17.

[21] Yarwood T. Infectious Diseases in North-East Australia; Burden of Disease & Health System Staffing. Tropical Public Health Services, Queensland Government. 2016.

[22] Australian Institute of Health and Welfare. Australia’s health 2018. Australia’s health series no. 16. AUS 221. 2018. https://www.aihw.gov.au/reports/australias-health/australias-health-2018/contents/table-of-contents.

[23] Australian Institute of Health and Welfare. Australian Burden of Disease Study: Impact and causes of illness and death in Aboriginal and Torres Strait Islander people 2011. Australian Burden of Disease Study series no. 6. Cat. no. BOD 7. 2016. https://www.aihw.gov.au/reports/burden-of-disease/illness-death-indigenous-australians/summary. 10.17061/phrp2741732 29114712

[24] Zhang X, Zhao Y, Guthridge S. Burden of Disease and Injury Study: impact and causes of illness, injury and death in the Northern Territory, 2004-2013. 2018. https://digitallibrary.health.nt.gov.au/prodjspui/bitstream/10137/7072/3/NT%20Burden%20of%20Disease%20and%20Injury%20Study%20-%20revised.pdf.

[25] Gibney KB, Cheng AC, Hall R et al Sociodemographic and geographical inequalities in notifiable infectious diseases in Australia: a retrospective analysis of 21 years of national disease surveillance data. Lancet Infect Dis 2017; 17: 86–97. 27789179 10.1016/S1473-3099(16)30309-7

[26] Tong SY, Varrone L, Chatfield MD et al Progressive increase in community-associated methicillin-resistant *Staphylococcus aureus* in Indigenous populations in northern Australia from 1993 to 2012. Epidemiol Infect 2015; 143: 1519–23. 25302939 10.1017/S0950268814002611 PMC9507213

[27] Wozniak TM, Cuningham W, Buchanan S et al Geospatial epidemiology of *Staphylococcus aureus* in a tropical setting: an enabling digital surveillance platform. Sci Rep 2020; 10: 13169. 32759953 10.1038/s41598-020-69312-4 PMC7406509

[28] StataCorp. Stata Statistical Software: Release 16. College Station, TX: StataCorp LLC, 2019.

[29] R Core Team. R: A Language and Environment for Statistical Computing. Vienna, Austria: R Foundation for Statistical Computing, 2020. https://www.R-project.org/.

[30] Meumann EM, Mitchell BG, McGregor A et al Urinary *Escherichia coli* antimicrobial susceptibility profiles and their relationship with community antibiotic use in Tasmania, Australia. Int J Antimicrob Agents 2015; 46: 389–93. 26187365 10.1016/j.ijantimicag.2015.05.015

[31] Simmering J, Cavanaugh J, Polgreen L et al Warmer weather as a risk factor for hospitalisations due to urinary tract infections. Epidemiol Infect 2018; 146: 386–93. 29307331 10.1017/S0950268817002965 PMC5808437

[32] Simmering JE, Tang F, Cavanaugh JE et al The increase in hospitalizations for urinary tract infections and the associated costs in the United States, 1998–2011. Open Forum Infect Dis 2017; 4: ofw281. 28480273 10.1093/ofid/ofw281 PMC5414046

[33] Yolbas I, Tekin R, Kelekci S et al Community-acquired urinary tract infections in children: pathogens, antibiotic susceptibility and seasonal changes. Eur Rev Med Pharmacol Sci 2013; 17: 971–6. 23640446

[34] Rosello A, Pouwels K, De Cellès MD et al Seasonality of urinary tract infections in the United Kingdom in different age groups: longitudinal analysis of The Health Improvement Network (THIN). Epidemiol Infect 2018; 146: 37–45. 29168442 10.1017/S095026881700259X PMC9134528

[35] Hawser SP, Bouchillon SK, Hoban DJ et al Emergence of high levels of extended-spectrum-β-lactamase-producing gram-negative bacilli in the Asia-pacific region: data from the Study for Monitoring Antimicrobial Resistance Trends (SMART) program, 2007. Antimicrob Agents Chemother 2009; 53: 3280–4. 19506060 10.1128/AAC.00426-09 PMC2715591

[36] Cheng AC, Turnidge J, Collignon P et al Control of fluoroquinolone resistance through successful regulation, Australia. Emerg Infect Dis 2012; 18: 1453–60. 22932272 10.3201/eid1809.111515 PMC3437704

[37] Uppala A, King EA, Patel D. Cefazolin versus fluoroquinolones for the treatment of community-acquired urinary tract infections in hospitalized patients. Eur J Clin Microbiol Infect Dis 2019; 38: 1533–8. 31114972 10.1007/s10096-019-03582-3

[38] Stewardson AJ, Vervoort J, Adriaenssens N et al Effect of outpatient antibiotics for urinary tract infections on antimicrobial resistance among commensal Enterobacteriaceae: a multinational prospective cohort study. Clin Microbiol Infect 2018; 24: 972–9. 29331548 10.1016/j.cmi.2017.12.026

[39] National Centre for Antimicrobial Stewardship and Australian Commission on Safety and Quality in Health Care. Antimicrobial prescribing practice in Australian hospitals: results of the 2018 Hospital National Antimicrobial Prescribing Survey. 2020. https://www.safetyandquality.gov.au/publications-and-resources/resource-library/antimicrobial-prescribing-practice-australian-hospitals-results-2018-hospital-national-antimicrobial-prescribing-survey-2.

[40] Cuningham W, Anderson L, Bowen AC et al Antimicrobial stewardship in remote primary healthcare across northern Australia. PeerJ 2020; 8: e9409. 32765965 10.7717/peerj.9409 PMC7382366

[41] Davidson L, Knight J, Bowen AC. Skin infections in Australian Aboriginal children: a narrative review. Med J Aust 2020; 212: 231–7. 31630410 10.5694/mja2.50361 PMC9543154

[42] The Australian Healthy Skin Consortium. National Healthy Skin Guideline for the Prevention, Treatment and Public Health Control of Impetigo, Scabies, Crusted Scabies and Tinea for Indigenous Populations and Communities in Australia (1st edition). 2018. https://infectiousdiseases.telethonkids.org.au/siteassets/media-images-wesfarmers-centre/national-healthy-skin-guideline-1st-ed.-2018.pdf.

[43] Stefani S, Chung DR, Lindsay JA et al Meticillin-resistant *Staphylococcus aureus* (MRSA): global epidemiology and harmonisation of typing methods. Int J Antimicrob Agents 2012; 39: 273–82. 22230333 10.1016/j.ijantimicag.2011.09.030

[44] Aguilar-Duran S, Horcajada JP, Sorli L et al Community-onset healthcare-related urinary tract infections: comparison with community and hospital-acquired urinary tract infections. J Infect 2012; 64: 478–83. 22285591 10.1016/j.jinf.2012.01.010

[45] Horcajada JP, Shaw E, Padilla B et al Healthcare-associated, community-acquired and hospital-acquired bacteraemic urinary tract infections in hospitalized patients: a prospective multicentre cohort study in the era of antimicrobial resistance. Clin Microbiol Infect 2012; 19: 962–8. 23279375 10.1111/1469-0691.12089

