# Supplementary material for: Antibiotic resistance in uropathogens across northern Australia 2007–20 and impact on treatment guidelines
Source: JAC Antimicrob Resist. 2021 Aug 14;3(3):dlab127. doi: 10.1093/jacamr/dlab127 (PMC8364662; doi:10.1093/jacamr/dlab127)
Supplement: dlab127_Supplementary_Data [file dlab127_supplementary_data.docx]

**Supplementary data**


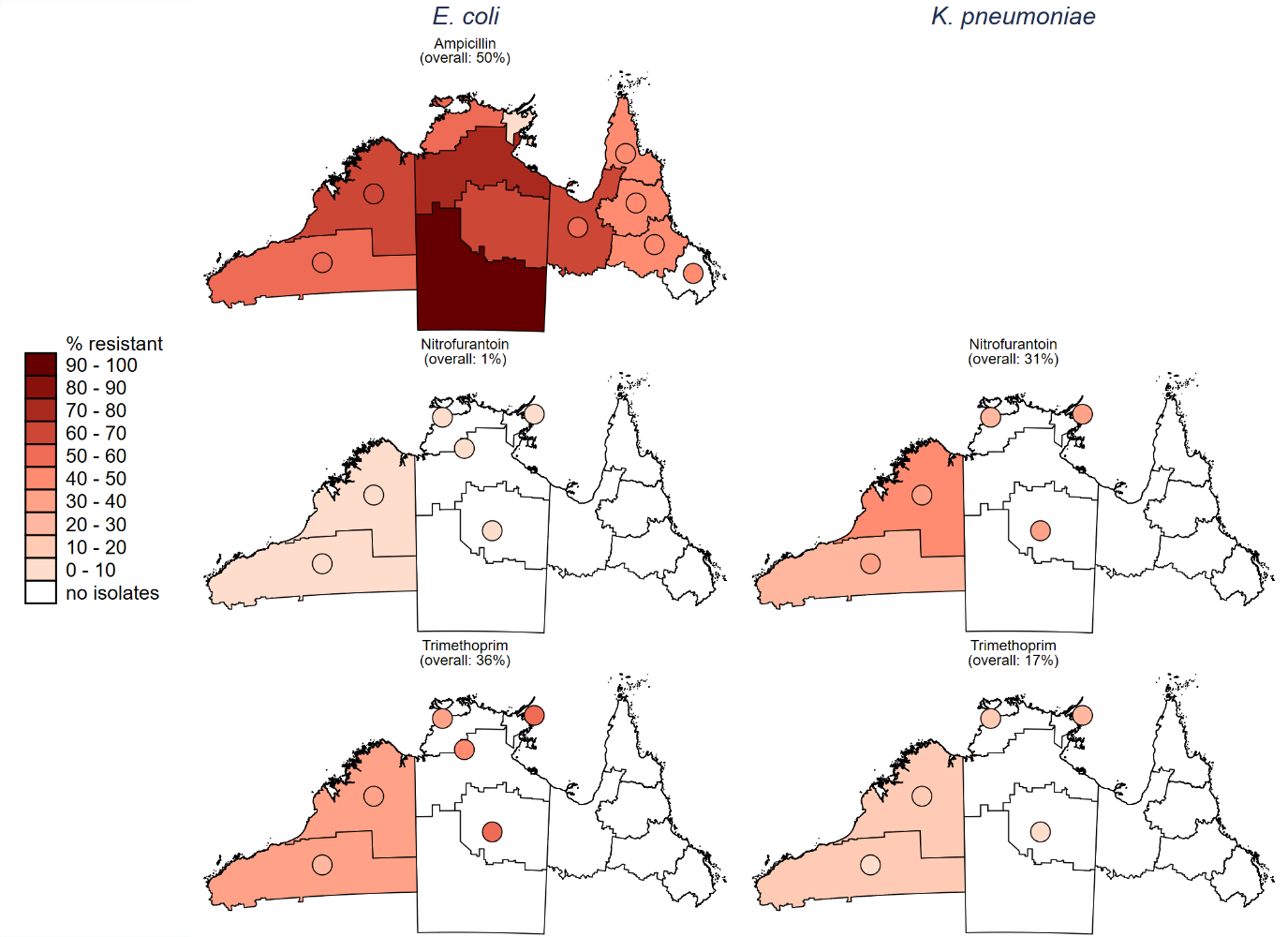


Figure S1. Proportion of isolates resistant to three antibiotics in 2019, by region and healthcare setting (community or hospital (displayed as circles)).

Regions (i.e. community healthcare facilities) with <30 isolates:

WA: Pilbara (*K. pneumoniae*/nitrofurantoin & trimethoprim)

NT: all regions (*E. coli*/ampicillin)

QLD: Cairns & Hinterland (*E. coli*/ampicillin); Townsville (*E. coli*/ampicillin)

Hospitals with <30 isolates:

WA: Pilbara (*K. pneumoniae*/nitrofurantoin & trimethoprim)

NT: East Arnhem (*K. pneumoniae*/nitrofurantoin & trimethoprim); Katherine (*E. coli*/nitrofurantoin & trimethoprim); Barkly (*K. pneumoniae*/nitrofurantoin & trimethoprim)


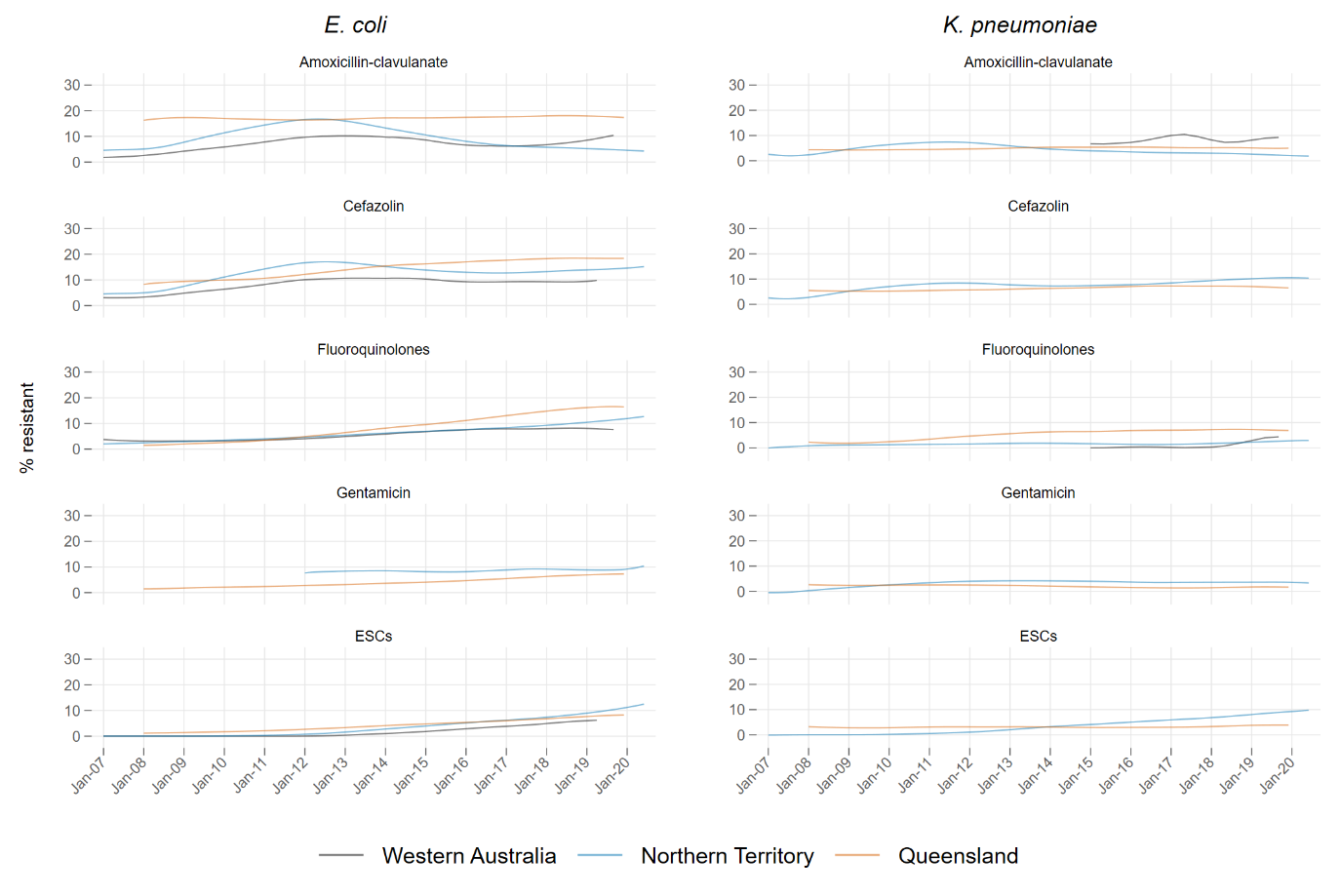


Figure S2. Proportion of isolates resistant to five antibiotics/antibiotic groups over time (smoothed using locally weighted regression), by jurisdiction (community & hospital isolates combined); ESCs: extended-spectrum cephalosporins (resistance to ceftriaxone or ceftazidime), Fluoroquinolones: resistance to ciprofloxacin or norfloxacin (only norfloxacin in WA hospitals).

Non-significant changes:

WA: *E. coli* & *K. pneumoniae*/amoxicillin-clavulanate & fluoroquinolones

QLD: *E. coli*/amoxicillin-clavulanate, *K. pneumoniae*/amoxicillin-clavulanate & ESCs

Significant decreases:

NT: *E. coli* & *K. pneumoniae*/amoxicillin-clavulanate

QLD: *K. pneumoniae*/gentamicin

WA: cefazolin & ESCs includes only community isolates; NT: gentamicin includes only hospital isolates for *E. coli*


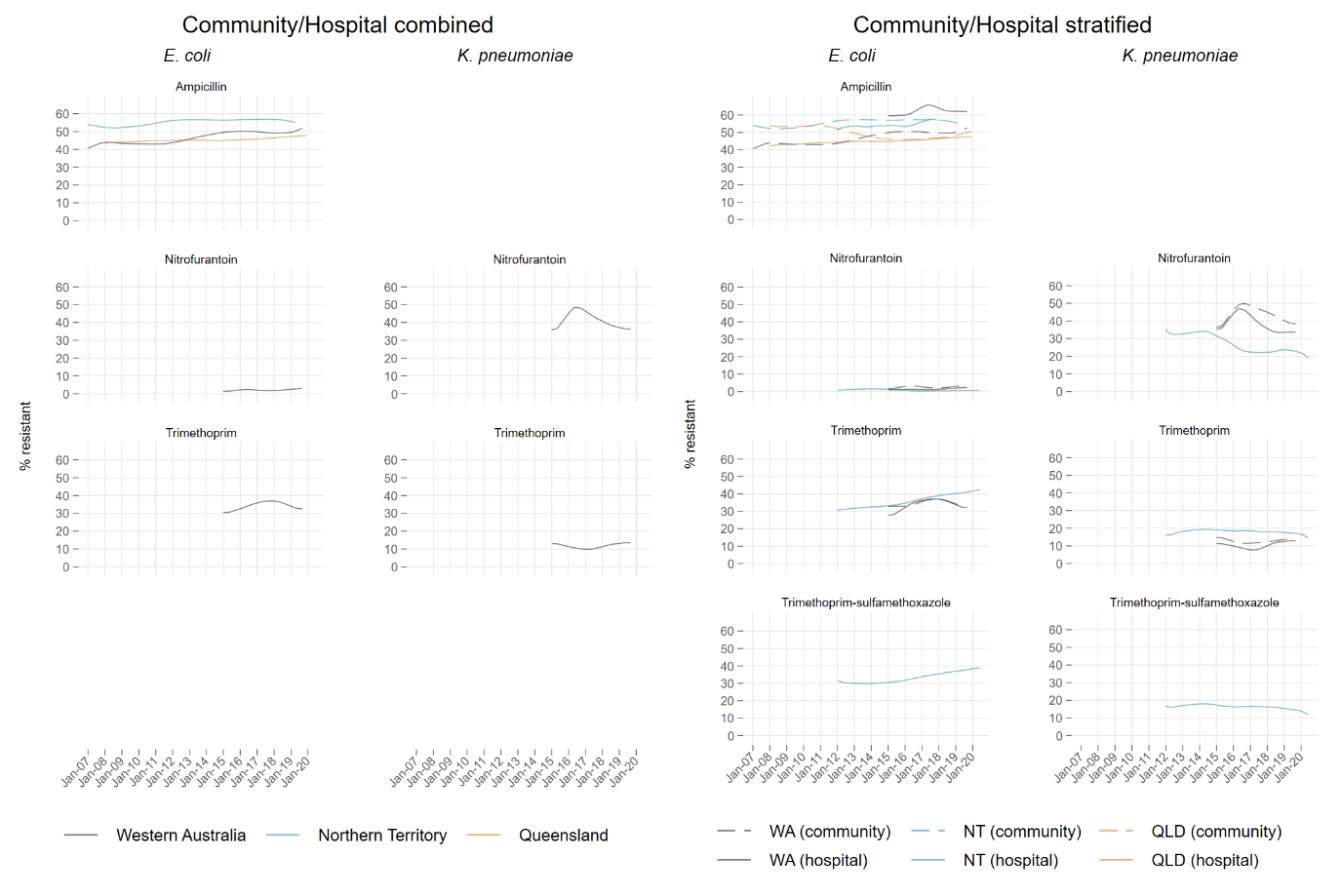


Figure S3. Proportion of isolates resistant to four antibiotics over time (smoothed using locally weighted regression).

Non-significant changes:

Community:

WA: *E. coli*/ampicillin & nitrofurantoin & trimethoprim, *K. pneumoniae*/nitrofurantoin & trimethoprim

Hospital:

WA: *E. coli*/ampicillin & nitrofurantoin & trimethoprim, *K. pneumoniae*/nitrofurantoin & trimethoprim

NT: *E. coli*/ampicillin, *K. pneumoniae*/trimethoprim & trimethoprim-sulfamethoxazole

Hospital & community combined:

WA: *E. coli*/ampicillin & nitrofurantoin & trimethoprim, *K. pneumoniae*/nitrofurantoin & trimethoprim

Significant decreases:

Community:

QLD: *E. coli*/ampicillin

Hospital:

NT: *E. coli* & *K. pneumoniae*/nitrofurantoin

Table S1. Number of isolates and percentage resistant in each jurisdiction, by organism, antibiotic and year.

| organism | antibiotic | year | WA | NT | FNQ |
| --- | --- | --- | --- | --- | --- |
| *E. coli* | Amoxicillin-clavulanate | 2007 | 255 (2.4%) | 3392 (4.5%) | NA |
| *E. coli* | Amoxicillin-clavulanate | 2008 | 311 (2.3%) | 3569 (6.2%) | 4638 (16.8%) |
| *E. coli* | Amoxicillin-clavulanate | 2009 | 248 (4.4%) | 3794 (5.2%) | 4884 (18.5%) |
| *E. coli* | Amoxicillin-clavulanate | 2010 | 147 (10.2%) | 3414 (14.3%) | 5267 (17.1%) |
| *E. coli* | Amoxicillin-clavulanate | 2011 | 132 (6.8%) | 3444 (19.5%) | 5646 (14.6%) |
| *E. coli* | Amoxicillin-clavulanate | 2012 | 176 (9.1%) | 5220 (17.8%) | 5799 (16.9%) |
| *E. coli* | Amoxicillin-clavulanate | 2013 | 280 (15%) | 5378 (15.6%) | 5773 (17%) |
| *E. coli* | Amoxicillin-clavulanate | 2014 | 293 (9.6%) | 5929 (13.6%) | 5823 (17%) |
| *E. coli* | Amoxicillin-clavulanate | 2015 | 1482 (14.4%) | 5874 (7%) | 6031 (18.1%) |
| *E. coli* | Amoxicillin-clavulanate | 2016 | 1617 (13.7%) | 6198 (6.5%) | 6173 (16.7%) |
| *E. coli* | Amoxicillin-clavulanate | 2017 | 1598 (13.5%) | 6373 (6.3%) | 6338 (18.5%) |
| *E. coli* | Amoxicillin-clavulanate | 2018 | 1629 (13.4%) | 5805 (5.3%) | 6451 (17.8%) |
| *E. coli* | Amoxicillin-clavulanate | 2019 | 1340 (16.3%) | 2953 (5.8%) | 6562 (17.9%) |
| *E. coli* | Amoxicillin-clavulanate | 2020 | NA | 730 (3.8%) | NA |
| *E. coli* | Cefazolin | 2007 | 257 (3.1%) | 3405 (4.4%) | NA |
| *E. coli* | Cefazolin | 2008 | 313 (2.9%) | 3579 (6.1%) | 3805 (9%) |
| *E. coli* | Cefazolin | 2009 | 248 (4.4%) | 3801 (4.9%) | 4572 (9.9%) |
| *E. coli* | Cefazolin | 2010 | 147 (11.6%) | 3421 (14.1%) | 5243 (10.1%) |
| *E. coli* | Cefazolin | 2011 | 132 (6.8%) | 3450 (18.9%) | 5633 (10.2%) |
| *E. coli* | Cefazolin | 2012 | 176 (9.1%) | 5233 (18.4%) | 5781 (12.6%) |
| *E. coli* | Cefazolin | 2013 | 280 (15.7%) | 5389 (16.6%) | 5736 (16%) |
| *E. coli* | Cefazolin | 2014 | 293 (9.9%) | 5948 (14.9%) | 5774 (15.9%) |
| *E. coli* | Cefazolin | 2015 | 256 (9%) | 5874 (11.8%) | 6004 (16.2%) |
| *E. coli* | Cefazolin | 2016 | 322 (10.2%) | 6196 (11.9%) | 6146 (17.4%) |
| *E. coli* | Cefazolin | 2017 | 340 (8.8%) | 6374 (13.8%) | 6313 (18.9%) |
| *E. coli* | Cefazolin | 2018 | 317 (9.5%) | 5807 (13%) | 6422 (18%) |
| *E. coli* | Cefazolin | 2019 | 114 (9.6%) | 2954 (14.2%) | 6525 (18.6%) |
| *E. coli* | Cefazolin | 2020 | NA | 729 (15.8%) | NA |
| *E. coli* | ESCs | 2007 | 257 (0%) | 3405 (0%) | NA |
| *E. coli* | ESCs | 2008 | 313 (0%) | 3583 (0.1%) | 4254 (1.4%) |
| *E. coli* | ESCs | 2009 | 248 (0%) | 3801 (0%) | 4813 (1.4%) |
| *E. coli* | ESCs | 2010 | 147 (0%) | 3421 (0%) | 5210 (1.9%) |
| *E. coli* | ESCs | 2011 | 132 (0%) | 3450 (0%) | 5594 (2.2%) |
| *E. coli* | ESCs | 2012 | 176 (0%) | 5234 (1.3%) | 5662 (2.8%) |
| *E. coli* | ESCs | 2013 | 280 (0%) | 5389 (1.4%) | 5563 (3.8%) |
| *E. coli* | ESCs | 2014 | 293 (1%) | 5948 (3.1%) | 5591 (4.2%) |
| *E. coli* | ESCs | 2015 | 256 (3.1%) | 5874 (5.5%) | 5823 (5.5%) |
| *E. coli* | ESCs | 2016 | 323 (2.8%) | 6199 (5.5%) | 5947 (5.4%) |
| *E. coli* | ESCs | 2017 | 340 (5%) | 6374 (6.4%) | 6128 (6.5%) |
| *E. coli* | ESCs | 2018 | 317 (6%) | 5807 (7.6%) | 6258 (7.2%) |
| *E. coli* | ESCs | 2019 | 114 (5.3%) | 2955 (8.2%) | 6316 (8.1%) |
| *E. coli* | ESCs | 2020 | NA | 730 (12.6%) | NA |
| *E. coli* | Fluoroquinolones | 2007 | 257 (2.7%) | 3405 (2.1%) | NA |
| *E. coli* | Fluoroquinolones | 2008 | 313 (3.2%) | 3583 (2.7%) | 4225 (1.7%) |
| *E. coli* | Fluoroquinolones | 2009 | 247 (2.8%) | 3802 (3.2%) | 4452 (2.2%) |
| *E. coli* | Fluoroquinolones | 2010 | 145 (4.8%) | 3416 (3.8%) | 4816 (3.1%) |
| *E. coli* | Fluoroquinolones | 2011 | 132 (3.8%) | 3449 (4.1%) | 5212 (3.1%) |
| *E. coli* | Fluoroquinolones | 2012 | 176 (3.4%) | 5229 (5.3%) | 5303 (5.5%) |
| *E. coli* | Fluoroquinolones | 2013 | 280 (4.6%) | 5389 (5.5%) | 5226 (7.9%) |
| *E. coli* | Fluoroquinolones | 2014 | 293 (6.5%) | 5949 (6.9%) | 5293 (8.2%) |
| *E. coli* | Fluoroquinolones | 2015 | 1487 (7.3%) | 5870 (7.5%) | 5383 (10.5%) |
| *E. coli* | Fluoroquinolones | 2016 | 1619 (8.1%) | 6199 (7.3%) | 5509 (11.7%) |
| *E. coli* | Fluoroquinolones | 2017 | 1597 (9%) | 6294 (9.1%) | 5707 (14.2%) |
| *E. coli* | Fluoroquinolones | 2018 | 1630 (9.5%) | 5807 (9.4%) | 5849 (16%) |
| *E. coli* | Fluoroquinolones | 2019 | 1349 (11.3%) | 2954 (10.8%) | 5959 (16.5%) |
| *E. coli* | Fluoroquinolones | 2020 | NA | 730 (13%) | NA |
| *E. coli* | Gentamicin | 2008 | NA | NA | 4638 (1.7%) |
| *E. coli* | Gentamicin | 2009 | NA | NA | 4883 (1.8%) |
| *E. coli* | Gentamicin | 2010 | NA | NA | 5263 (2.8%) |
| *E. coli* | Gentamicin | 2011 | NA | NA | 5642 (2%) |
| *E. coli* | Gentamicin | 2012 | NA | 1018 (7.8%) | 5798 (2.8%) |
| *E. coli* | Gentamicin | 2013 | NA | 981 (8.9%) | 5762 (3.8%) |
| *E. coli* | Gentamicin | 2014 | NA | 1123 (9.1%) | 5787 (3.6%) |
| *E. coli* | Gentamicin | 2015 | NA | 1211 (8.1%) | 6020 (4.3%) |
| *E. coli* | Gentamicin | 2016 | NA | 1275 (7.6%) | 6169 (4.9%) |
| *E. coli* | Gentamicin | 2017 | NA | 1535 (10.2%) | 6335 (6.3%) |
| *E. coli* | Gentamicin | 2018 | NA | 1302 (9.5%) | 6446 (6.3%) |
| *E. coli* | Gentamicin | 2019 | NA | 1300 (8.2%) | 6561 (7.4%) |
| *E. coli* | Gentamicin | 2020 | NA | 730 (9.9%) | NA |
| *K. pneumoniae* | Amoxicillin-clavulanate | 2007 | 30 (3.3%) | 272 (2.2%) | NA |
| *K. pneumoniae* | Amoxicillin-clavulanate | 2008 | 43 (0%) | 396 (1.8%) | 847 (4.4%) |
| *K. pneumoniae* | Amoxicillin-clavulanate | 2009 | 25 (0%) | 447 (4.3%) | 782 (3.8%) |
| *K. pneumoniae* | Amoxicillin-clavulanate | 2010 | 15 (0%) | 439 (11.2%) | 943 (4.1%) |
| *K. pneumoniae* | Amoxicillin-clavulanate | 2011 | 12 (0%) | 584 (8.6%) | 941 (5.1%) |
| *K. pneumoniae* | Amoxicillin-clavulanate | 2012 | 26 (7.7%) | 841 (5.5%) | 1047 (4.8%) |
| *K. pneumoniae* | Amoxicillin-clavulanate | 2013 | 36 (0%) | 836 (5.6%) | 983 (4.8%) |
| *K. pneumoniae* | Amoxicillin-clavulanate | 2014 | 32 (6.2%) | 924 (4.1%) | 1027 (5.6%) |
| *K. pneumoniae* | Amoxicillin-clavulanate | 2015 | 196 (6.1%) | 982 (3.3%) | 1172 (6%) |
| *K. pneumoniae* | Amoxicillin-clavulanate | 2016 | 247 (6.9%) | 968 (2.8%) | 1222 (4.2%) |
| *K. pneumoniae* | Amoxicillin-clavulanate | 2017 | 223 (9.9%) | 834 (4%) | 1266 (6.1%) |
| *K. pneumoniae* | Amoxicillin-clavulanate | 2018 | 230 (6.5%) | 808 (2.1%) | 1354 (4.9%) |
| *K. pneumoniae* | Amoxicillin-clavulanate | 2019 | 192 (8.9%) | 473 (3.8%) | 1287 (5.2%) |
| *K. pneumoniae* | Amoxicillin-clavulanate | 2020 | NA | 144 (1.4%) | NA |
| *K. pneumoniae* | Cefazolin | 2007 | 30 (3.3%) | 276 (2.5%) | NA |
| *K. pneumoniae* | Cefazolin | 2008 | 43 (0%) | 398 (1.8%) | 710 (5.5%) |
| *K. pneumoniae* | Cefazolin | 2009 | 25 (0%) | 450 (5.8%) | 732 (4.2%) |
| *K. pneumoniae* | Cefazolin | 2010 | 15 (0%) | 442 (11.5%) | 938 (5.5%) |
| *K. pneumoniae* | Cefazolin | 2011 | 12 (0%) | 585 (8.7%) | 940 (6%) |
| *K. pneumoniae* | Cefazolin | 2012 | 26 (7.7%) | 842 (6.9%) | 1042 (6.5%) |
| *K. pneumoniae* | Cefazolin | 2013 | 36 (0%) | 840 (8.1%) | 978 (5.1%) |
| *K. pneumoniae* | Cefazolin | 2014 | 33 (6.1%) | 930 (6.5%) | 1009 (6.6%) |
| *K. pneumoniae* | Cefazolin | 2015 | 20 (0%) | 984 (7.3%) | 1167 (7.1%) |
| *K. pneumoniae* | Cefazolin | 2016 | 37 (2.7%) | 968 (7.7%) | 1215 (7%) |
| *K. pneumoniae* | Cefazolin | 2017 | 36 (5.6%) | 834 (8.9%) | 1261 (7.3%) |
| *K. pneumoniae* | Cefazolin | 2018 | 27 (3.7%) | 808 (8.7%) | 1352 (7.2%) |
| *K. pneumoniae* | Cefazolin | 2019 | 8 (0%) | 473 (13.1%) | 1286 (6.9%) |
| *K. pneumoniae* | Cefazolin | 2020 | NA | 144 (9%) | NA |
| *K. pneumoniae* | ESCs | 2007 | 30 (0%) | 276 (0%) | NA |
| *K. pneumoniae* | ESCs | 2008 | 43 (0%) | 399 (0%) | 796 (3.1%) |
| *K. pneumoniae* | ESCs | 2009 | 25 (0%) | 450 (0.2%) | 780 (2.1%) |
| *K. pneumoniae* | ESCs | 2010 | 15 (0%) | 442 (0%) | 938 (2.8%) |
| *K. pneumoniae* | ESCs | 2011 | 12 (0%) | 585 (0%) | 939 (3.7%) |
| *K. pneumoniae* | ESCs | 2012 | 26 (0%) | 842 (2%) | 1037 (4%) |
| *K. pneumoniae* | ESCs | 2013 | 36 (0%) | 840 (2.1%) | 967 (2.3%) |
| *K. pneumoniae* | ESCs | 2014 | 33 (0%) | 930 (3%) | 1000 (3.6%) |
| *K. pneumoniae* | ESCs | 2015 | 20 (0%) | 984 (6.3%) | 1151 (3%) |
| *K. pneumoniae* | ESCs | 2016 | 37 (0%) | 968 (4.9%) | 1199 (2.3%) |
| *K. pneumoniae* | ESCs | 2017 | 36 (0%) | 834 (5.4%) | 1251 (3.1%) |
| *K. pneumoniae* | ESCs | 2018 | 27 (0%) | 808 (6.3%) | 1336 (3.6%) |
| *K. pneumoniae* | ESCs | 2019 | 8 (0%) | 473 (10.4%) | 1280 (3.9%) |
| *K. pneumoniae* | ESCs | 2020 | NA | 144 (8.3%) | NA |
| *K. pneumoniae* | Fluoroquinolones | 2007 | 30 (0%) | 276 (0.4%) | NA |
| *K. pneumoniae* | Fluoroquinolones | 2008 | 43 (0%) | 399 (1.3%) | 794 (1.9%) |
| *K. pneumoniae* | Fluoroquinolones | 2009 | 25 (0%) | 450 (1.8%) | 726 (1.4%) |
| *K. pneumoniae* | Fluoroquinolones | 2010 | 15 (0%) | 442 (1.4%) | 866 (2.2%) |
| *K. pneumoniae* | Fluoroquinolones | 2011 | 12 (0%) | 584 (0.5%) | 878 (4.4%) |
| *K. pneumoniae* | Fluoroquinolones | 2012 | 26 (3.8%) | 842 (2.4%) | 972 (5.7%) |
| *K. pneumoniae* | Fluoroquinolones | 2013 | 36 (0%) | 840 (1.8%) | 894 (5.7%) |
| *K. pneumoniae* | Fluoroquinolones | 2014 | 33 (0%) | 930 (2.2%) | 942 (7%) |
| *K. pneumoniae* | Fluoroquinolones | 2015 | 196 (0%) | 983 (1.5%) | 1079 (6.9%) |
| *K. pneumoniae* | Fluoroquinolones | 2016 | 247 (0.8%) | 968 (1%) | 1113 (5.8%) |
| *K. pneumoniae* | Fluoroquinolones | 2017 | 224 (0%) | 827 (1.2%) | 1134 (7.8%) |
| *K. pneumoniae* | Fluoroquinolones | 2018 | 230 (0.4%) | 807 (1.5%) | 1231 (7.2%) |
| *K. pneumoniae* | Fluoroquinolones | 2019 | 192 (4.2%) | 473 (3.2%) | 1202 (7.1%) |
| *K. pneumoniae* | Fluoroquinolones | 2020 | NA | 144 (2.1%) | NA |
| *K. pneumoniae* | Gentamicin | 2007 | NA | 175 (0%) | NA |
| *K. pneumoniae* | Gentamicin | 2008 | NA | 219 (0%) | 847 (2.6%) |
| *K. pneumoniae* | Gentamicin | 2009 | NA | 224 (2.7%) | 780 (1.5%) |
| *K. pneumoniae* | Gentamicin | 2010 | NA | 244 (3.7%) | 942 (2.5%) |
| *K. pneumoniae* | Gentamicin | 2011 | NA | 383 (3.9%) | 940 (3.3%) |
| *K. pneumoniae* | Gentamicin | 2012 | NA | 768 (3.9%) | 1046 (2.5%) |
| *K. pneumoniae* | Gentamicin | 2013 | NA | 720 (4.9%) | 980 (1.9%) |
| *K. pneumoniae* | Gentamicin | 2014 | NA | 806 (3.8%) | 1016 (2.3%) |
| *K. pneumoniae* | Gentamicin | 2015 | NA | 908 (3.6%) | 1171 (1.9%) |
| *K. pneumoniae* | Gentamicin | 2016 | NA | 901 (3.9%) | 1222 (0.9%) |
| *K. pneumoniae* | Gentamicin | 2017 | NA | 773 (3%) | 1260 (1.3%) |
| *K. pneumoniae* | Gentamicin | 2018 | NA | 772 (3.8%) | 1353 (1.9%) |
| *K. pneumoniae* | Gentamicin | 2019 | NA | 460 (4.3%) | 1286 (1.7%) |
| *K. pneumoniae* | Gentamicin | 2020 | NA | 144 (2.8%) | NA |

Table S2. Linear regression, percent resistant by month^.

| setting | organism_jurisdiction_antibiotic | month^ coefficient | constant | N |
| --- | --- | --- | --- | --- |
| Overall | Ecoli_FNQ_Amox_clav | 0.00907 (1.82) | 11.37*** (3.57) | 144 |
|  | Ecoli_FNQ_Cefazolin | 0.0838*** (17.62) | -39.87*** (-12.92) | 144 |
|  | Ecoli_FNQ_Fluoro | 0.123*** (37.97) | -71.06*** (-33.89) | 144 |
|  | Ecoli_FNQ_Gent | 0.0425*** (19.62) | -23.54*** (-16.76) | 144 |
|  | Ecoli_FNQ_ESCs | 0.0527*** (23.04) | -29.93*** (-20.17) | 144 |
|  |  | | | |
|  | Ecoli_NT_Amox_clav | -0.0279** (-2.87) | 27.55*** (4.39) | 162 |
|  | Ecoli_NT_Cefazolin | 0.0452*** (5.63) | -16.49** (-3.18) | 162 |
|  | Ecoli_NT_Fluoro | 0.0612*** (23.45) | -33.23*** (-19.71) | 162 |
|  | Ecoli_NT_ESCs | 0.0731*** (25.52) | -43.69*** (-23.59) | 162 |
|  |  | | | |
|  | Ecoli_WA_Amox_clav^ | 1.381 (1.73) | -2768.2 (-1.72) | 9 |
|  | Ecoli_WA_Fluoro^ | 0.721 (1.57) | -1442.5 (-1.55) | 9 |
|  |  | | | |
|  | Kpneu_FNQ_Amox_clav | 0.00858 (1.84) | -0.609 (-0.20) | 144 |
|  | Kpneu_FNQ_Cefazolin | 0.0190*** (3.69) | -6.040 (-1.81) | 144 |
|  | Kpneu_FNQ_Fluoro | 0.0468*** (8.36) | -25.03*** (-6.89) | 144 |
|  | Kpneu_FNQ_Gent | -0.00927** (-2.96) | 8.019*** (3.95) | 144 |
|  | Kpneu_FNQ_ESCs | 0.00465 (1.19) | 0.121 (0.05) | 144 |
|  |  | | | |
|  | Kpneu_NT_Amox_clav | -0.0170** (-2.69) | 15.39*** (3.75) | 162 |
|  | Kpneu_NT_Cefazolin | 0.0389*** (5.51) | -17.49*** (-3.84) | 162 |
|  | Kpneu_NT_Fluoro | 0.00797* (2.34) | -3.586 (-1.63) | 162 |
|  | Kpneu_NT_Gent | 0.0181*** (3.78) | -8.463** (-2.74) | 161 |
|  | Kpneu_NT_ESCs | 0.0654*** (14.38) | -38.79*** (-13.20) | 162 |
|  |  | | | |
|  | Kpneu_WA_Amox_clav^ | 0.368 (0.53) | -734.2 (-0.53) | 5 |
|  | Kpneu_WA_Fluoro^ | 0.862 (1.93) | -1737.3 (-1.93) | 5 |
| Community | Ecoli_FNQ_Amox_clav | -0.00773 (-0.66) | 21.07** (2.76) | 144 |
|  | Ecoli_FNQ_Cefazolin | 0.121*** (9.19) | -61.37*** (-7.20) | 144 |
|  | Ecoli_FNQ_Fluoro | 0.115*** (11.05) | -66.51*** (-9.83) | 143 |
|  | Ecoli_FNQ_Gent | 0.0348*** (5.41) | -19.30*** (-4.62) | 144 |
|  | Ecoli_FNQ_ESCs | 0.0252*** (5.43) | -14.5*** (-4.81) | 144 |
|  |  | | | |
|  | Ecoli_NT_Amox_clav | -0.0198 (-1.56) | 22.96** (2.83) | 148 |
|  | Ecoli_NT_Cefazolin | 0.0504*** (4.99) | -19.48** (-3.02) | 148 |
|  | Ecoli_NT_Fluoro | 0.0524*** (19.67) | -28.07*** (-16.48) | 148 |
|  | Ecoli_NT_ESCs | 0.0578*** (19.44) | -34.62*** (-18.23) | 148 |
|  |  | | | |
|  | Ecoli_WA_Amox_clav^ | 1.484 (1.84) | -2975.4 (-1.83) | 9 |
|  | Ecoli_WA_Cefazolin^ | 1.776 (1.31) | -3563.5 (-1.30) | 7 |
|  | Ecoli_WA_Fluoro^ | 0.842 (1.96) | -1686 (-1.95) | 9 |
|  | Ecoli_WA_ESCs^ | 1.359 (1.21) | -2732.1 (-1.21) | 7 |
|  |  | | | |
|  | Kpneu_FNQ_Amox_clav | 0.105 (1.74) | -58.73 (-1.54) | 11 |
|  | Kpneu_FNQ_Cefazolin | 0.216** (3.74) | -129.9** (-3.57) | 11 |
|  | Kpneu_FNQ_ESCs | -0.00855 (-0.19) | 6.43 (0.23) | 9 |
|  |  |  |  |  |
|  | Kpneu_NT_Amox_clav | -0.0153 (-1.90) | 14.48** (2.81) | 148 |
|  | Kpneu_NT_Cefazolin | 0.0247** (2.85) | -9.310 (-1.68) | 148 |
|  | Kpneu_NT_Fluoro | -0.00139 (-0.47) | 1.821 (0.97) | 148 |
|  | Kpneu_NT_Gent | 0.0154** (2.98) | -7.339* (-2.21) | 147 |
|  | Kpneu_NT_ESCs | 0.0468*** (9.38) | -28.13*** (-8.82) | 148 |
|  |  | | | |
|  | Kpneu_WA_Amox_clav^ | 1.400* (4.05) | -2814.2* (-4.04) | 5 |
|  | Kpneu_WA_Fluoro^ | 0.999 (2.05) | -2014.4 (-2.05) | 5 |
| Hospital | Ecoli_FNQ_Amox_clav | 0.0104* (1.98) | 10.59** (3.10) | 144 |
|  | Ecoli_FNQ_Cefazolin | 0.0816*** (16.78) | -38.56*** (-12.23) | 144 |
|  | Ecoli_FNQ_Fluoro | 0.123*** (37.24) | -71.34*** (-33.25) | 144 |
|  | Ecoli_FNQ_Gent | 0.0428*** (19.14) | -23.66*** (-16.33) | 144 |
|  | Ecoli_FNQ_ESCs | 0.0539*** (22.76) | -30.56*** (-19.89) | 144 |
|  |  | | | |
|  | Ecoli_NT_Amox_clav | -0.0313** (-2.92) | 28.49*** (3.94) | 102 |
|  | Ecoli_NT_Cefazolin | 0.0290* (2.32) | -6.575 (-0.78) | 102 |
|  | Ecoli_NT_Fluoro | 0.0335** (3.17) | -12.59 (-1.77) | 102 |
|  | Ecoli_NT_Gent | 0.00992 (1.01) | 1.929 (0.29) | 102 |
|  | Ecoli_NT_ESCs | 0.0404*** (4.57) | -18.78** (-3.15) | 102 |
|  |  | | | |
|  | Ecoli_WA_Amox_clav^ | 0.772 (1.70) | -1542.4 (-1.69) | 5 |
|  | Ecoli_WA_Fluoro^ | 1.146* (5.62) | -2303.6* (-5.60) | 5 |
|  |  | | | |
|  | Kpneu_FNQ_Amox_clav | 0.00674 (1.39) | 0.568 (0.18) | 144 |
|  | Kpneu_FNQ_Cefazolin | 0.0148** (2.73) | -3.295 (-0.94) | 144 |
|  | Kpneu_FNQ_Fluoro | 0.0458*** (7.7) | -24.24*** (-6.28) | 144 |
|  | Kpneu_FNQ_Gent | -0.0115*** (-3.40) | 9.589*** (4.36) | 144 |
|  | Kpneu_FNQ_ESCs | 0.00285 (0.68) | 1.445 (0.53) | 144 |
|  |  | | | |
|  | Kpneu_NT_Amox_clav | 0.00293 (0.22) | 1.373 (0.15) | 102 |
|  | Kpneu_NT_Cefazolin | 0.0110 (0.49) | 3.460 (0.23) | 102 |
|  | Kpneu_NT_Fluoro | -0.0326* (-2.30) | 25.49** (2.66) | 102 |
|  | Kpneu_NT_Gent | -0.0580*** (-3.70) | 44.86*** (4.24) | 102 |
|  | Kpneu_NT_ESCs | 0.00758 (0.36) | 3.795 (0.26) | 102 |
|  |  | | | |
|  | Kpneu_WA_Amox_clav^ | -0.899 (-0.68) | 1821.3 (0.69) | 5 |
|  | Kpneu_WA_Fluoro^ | 0.706 (1.73) | -1423.1 (-1.73) | 5 |

^by year for WA data

t-statistic in parentheses: * p < 0.05, ** p < 0.01, *** p < 0.00

Table S3. Number of isolates and percentage resistant in each jurisdiction, by organism, antibiotic and healthcare setting of specimen collection.

| organism | antibiotic | setting | WA | NT | FNQ |
| --- | --- | --- | --- | --- | --- |
| *E. coli* | Amoxicillin-clavulanate | All | 9508 (12.8%) | 62073 (9.7%) | 69385 (17.3%) |
| *E. coli* | Amoxicillin-clavulanate | Community | 6545 (12%) | 51594 (10.2%) | 4962 (16.1%) |
| *E. coli* | Amoxicillin-clavulanate | Hospital | 2963 (14.7%) | 10479 (7.4%) | 64423 (17.3%) |
| *E. coli* | Cefazolin | All | NA | 62160 (12.9%) | 67954 (14.8%) |
| *E. coli* | Cefazolin | Community | 3195 (8.5%) | 51687 (12.9%) | 4945 (16.5%) |
| *E. coli* | Cefazolin | Hospital | NA | 10473 (13.1%) | 63009 (14.7%) |
| *E. coli* | ESCs | All | NA | 62170 (3.5%) | 67159 (4.4%) |
| *E. coli* | ESCs | Community | 3196 (1.9%) | 51694 (2.5%) | 4589 (1.8%) |
| *E. coli* | ESCs | Hospital | NA | 10476 (8.6%) | 62570 (4.6%) |
| *E. coli* | Fluoroquinolones | All | 9525 (8%) | 62076 (6.4%) | 62934 (8.8%) |
| *E. coli* | Fluoroquinolones | Community | 6548 (8%) | 51601 (5.6%) | 3699 (7.8%) |
| *E. coli* | Fluoroquinolones | Hospital | 2977 (8.1%) | 10475 (10.1%) | 59235 (8.9%) |
| *E. coli* | Gentamicin | All | NA | NA | 69304 (4.1%) |
| *E. coli* | Gentamicin | Community | NA | NA | 4948 (3.2%) |
| *E. coli* | Gentamicin | Hospital | NA | 10475 (8.8%) | 64356 (4.2%) |
| *K. pneumoniae* | Amoxicillin-clavulanate | All | 1307 (6.7%) | 8948 (4.4%) | 12871 (5%) |
| *K. pneumoniae* | Amoxicillin-clavulanate | Community | 870 (6.2%) | 6577 (4.7%) | 791 (5.1%) |
| *K. pneumoniae* | Amoxicillin-clavulanate | Hospital | 437 (7.8%) | 2371 (3.4%) | 12080 (5%) |
| *K. pneumoniae* | Cefazolin | All | NA | 8974 (7.7%) | 12630 (6.4%) |
| *K. pneumoniae* | Cefazolin | Community | 348 (2.6%) | 6603 (6.5%) | 784 (6.5%) |
| *K. pneumoniae* | Cefazolin | Hospital | NA | 2371 (11.1%) | 11846 (6.4%) |
| *K. pneumoniae* | ESCs | All | NA | 8975 (3.7%) | 12674 (3.2%) |
| *K. pneumoniae* | ESCs | Community | 348 (0%) | 6604 (1.8%) | 764 (0.9%) |
| *K. pneumoniae* | ESCs | Hospital | NA | 2371 (9%) | 11910 (3.3%) |
| *K. pneumoniae* | Fluoroquinolones | All | 1309 (0.9%) | 8965 (1.6%) | 11831 (5.5%) |
| *K. pneumoniae* | Fluoroquinolones | Community | 871 (1%) | 6594 (1%) | 583 (2.7%) |
| *K. pneumoniae* | Fluoroquinolones | Hospital | 438 (0.7%) | 2371 (3.4%) | 11248 (5.7%) |
| *K. pneumoniae* | Gentamicin | All | NA | 7497 (3.6%) | 12843 (2%) |
| *K. pneumoniae* | Gentamicin | Community | NA | 5126 (2.7%) | 786 (0.5%) |
| *K. pneumoniae* | Gentamicin | Hospital | NA | 2371 (5.6%) | 12057 (2.1%) |

Table S4. Linear regression, monthly^ percentage resistance in community isolates vs hospital isolates.

| jurisdiction_organism_antibiotic | coefficient (95% CI) | model fit (p-value), R^2^ | spearman correlation |
| --- | --- | --- | --- |
| FNQ_Ecoli_Amox_clav | 0.12 (-0.25, 0.49) | 0.52, 0.003 | 0.09 |
| FNQ_Ecoli_Cefazolin | 1.17 (0.91, 1.44)** | <0.001, 0.35 | 0.62* |
| FNQ_Ecoli_Fluoro | 0.87 (0.71, 1.03)** | <0.001, 0.45 | 0.68* |
| FNQ_Ecoli_Gent | 0.68 (0.43, 0.94)** | <0.001, 0.17 | 0.35* |
| FNQ_Ecoli_ESCs | 0.44 (0.29, 0.59)** | <0.001, 0.19 | 0.42* |
|  | | | |
| NT_Ecoli_Amox_clav | 0.10 (-0.32, 0.53) | 0.63, 0.003 | 0.04 |
| NT_Ecoli_Cefazolin | -0.04 (-0.25, 0.18) | 0.74, 0.001 | -0.07 |
| NT_Ecoli_Fluoro | 0.17 (0.03, 0.31)* | 0.02, 0.06 | 0.21 |
| NT_Ecoli_ESCs | 0.28 (0.05, 0.52)* | 0.02, 0.06 | 0.26* |
|  | | | |
| WA_Ecoli_Amox_clav^ | -0.50 (-1.32, 0.33) | 0.15, 0.55 | -0.90* |
| WA_Ecoli_Fluoro^ | 0.73 (0.13, 1.32)* | 0.03, 0.83 | 0.90* |
|  | | | |
| FNQ_Kpneu_Amox_clav | 2.74 (0.13, 5.35) | 0.04, 0.39 | 0.38 |
| FNQ_Kpneu_Cefazolin | 0.29 (-3.16, 3.74) | 0.85, 0.004 | -0.01 |
| FNQ_Kpneu_ESCs | 0.79 (-0.75, 2.34) | 0.26, 0.17 | 0.55 |
|  | | | |
| NT_Kpneu_Amox_clav | -0.17 (-0.35, 0.004) | 0.06, 0.04 | -0.18 |
| NT_Kpneu_Cefazolin | -0.02 (-0.15, 0.11) | 0.73, 0.001 | 0.0003 |
| NT_Kpneu_Fluoro | 0.05 (-0.01, 0.12) | 0.09, 0.03 | 0.14 |
| NT_Kpneu_Gent | 0.06 (-0.04, 0.15) | 0.24, 0.02 | 0.07 |
| NT_Kpneu_ESCs | 0.02 (-0.12, 0.15) | 0.75, 0.001 | 0.12 |
|  | | | |
| WA_Kpneu_Amox_clav^ | -0.11 (-1.23, 1.01) | 0.77, 0.03 | -0.2 |
| WA_Kpneu_Fluoro^ | 1.28 (0.79, 1.78)* | <0.01, 0.96 | 0.73 |

^yearly for WA data

*p<0.05; **p<0.001
